# Adequacy of knowledge of new medical graduates about diagnosis and management of children and adolescents with type 1 diabetes in a developing country

**DOI:** 10.1186/s12909-023-04234-z

**Published:** 2023-04-12

**Authors:** Abeer Alassaf, Lobna Gharaibeh, Lina Abuna’meh, Rasha Odeh

**Affiliations:** 1 Department of Pediatrics, University of Jordan, Queen Rania Street, Amman, 11942 Jordan; 2 Pharmacological and Diagnostic Research Center, Faculty of Pharmacy, Al-Ahliyya Amman University, Amman, Jordan

**Keywords:** Knowledge, New graduates, Type 1 diabetes, Jordan

## Abstract

**Background:**

Knowledge of diabetes by the graduate physicians had been reported to be deficient in many aspects of diagnosis and management of type 1 diabetes (T1D). This will reflect on patient care and quality of health services especially in limited-resources countries. Our aim was to assess knowledge of basic management of T1D in new medical graduates in Jordan.

**Methods:**

A questionnaire was developed to collect information concerning demographics and knowledge and was distributed in paper form and online using google forms. The knowledge was assessed using 28 questions on different aspects of the disease.

**Results:**

A total of 358 new medicine graduates responded to the survey and female respondents were significantly higher than male respondents. Average number of lectures concerning diabetes during the medical school years was 3.92 ± 1.37. High knowledge scores were on pathophysiology of T1D, hypoglycemia, and certain aspects of diabetic ketoacidosis. Female gender, higher number of persons with T1D the participant had encountered during medical school, and good or excellent expected degree of self-knowledge of diabetes were associated with high knowledge score, p values = 0.01, 0.009, and < 0.001, respectively. Female gender and good or excellent expected degree of knowledge of diabetes predicted high knowledge score, p value = 0.008, and < 0.001, respectively.

**Conclusion:**

Gaps in knowledge of new medical graduates in certain T1D subjects exist. This can be corrected by many strategies including changes in curricula, elective courses, more clinical exposure, and interprofessional education. These measures must be evaluated for their short and long-term benefits.

**Supplementary Information:**

The online version contains supplementary material available at 10.1186/s12909-023-04234-z.

## Introduction

Type 1 diabetes (T1D) represents about 2% of the estimated global total cases of diabetes [1]. The incidence and prevalence of T1D is increasing. A recent meta-analysis revealed a global prevalence of 9.5% and the incidence was 15 per 100,000 population [2]. The Middle East and North Africa region estimated prevalence of T1D in the age group 0–19 years in 2021 of 192,500 cases and incidence of 25,000 [3].

Type 1 diabetes generally starts during childhood with a long-term progression through adulthood and cumulative costs over time. The economic burden includes the cost of treatment, disease complications, and quality-of-life effects. Data related to the burden of T1D on the health sector and economy is scarce and mistakenly reported together with the costs of type 2 diabetes. Tao et al. showed annual T1D costs in the United States (US) of around 14.4–14.9 billion US dollars [4].

Furthermore, diabetic ketoacidosis (DKA) that is mainly associated with T1D [5, 6] is a severe complication that demands hospitalization and is associated with poor metabolic control [7]. There is no nationwide study on incidence of DKA in Jordan, but one study showed percentage of 31.7% of DKA at diagnosis of T1D in children and adolescents less than age of 16 years [8].

Since the acute and chronic complications of diabetes, in both types 1 and 2, can be prevented or delayed by appropriate intervention, comprehensive and sufficient knowledge by physicians about all aspects of diabetes management is of great importance. Zgibor et al. revealed that persons with type 1 diabetes who received specialist care had a lower HbA1c level compared to general physician care (9.7 vs. 10.3%; P = 0.0006) [9].

Many reports have shown that the lack of sufficient and appropriate knowledge by the treating physicians concerning the management of diabetes [10, 11]. In addition, trainee doctors are not fully confident in managing and optimizing glycemic control for their patients [12].

Preparing future physicians for the great task that lays ahead starts with medical education during undergraduate years. Satisfactory knowledge of diabetes diagnosis, management, and patient education is crucial and pivotal. Studies showed that medical students possessed shortcomings in certain aspects of diabetes management that included insulin treatment options [13], diabetic dietary counseling [14], and management of hypoglycemia and perioperative diabetes management guidelines [15]. Therefore, appropriate knowledge of the diagnosis and accurate management of T1D is of great importance.

Most children and adolescents with T1D in Jordan, which was classified recently as upper-middle-income country [16]; are seen by physicians other than pediatric endocrinologists as the number of the latter is scarce in the country and located mainly in the capital city. Assessment of knowledge of T1D for new medical graduates is necessary to identify flaws, weaknesses, and defects. There is paucity of studies concerning this subject all over the world, and such studies were not done previously in Jordan. The aim of our study was to assess knowledge of diagnosis and basic management of persons with T1D in new medical graduates in Jordan. Based on the findings, recommendations that involve modifications in the curriculum of medical schools and strategies to improve basic knowledge and clinical training concerning T1D would be offered.

## Methods

This is a cross-sectional questionnaire-based study, conducted from the 1st of August 2021 to the 31st of October, 2021. Jordan University Hospital Institutional Review Board (no.: 2021/14) approval was obtained before commencing the study. Recent medical school graduates from Jordanian universities that grant a bachelor degree in medicine; were approached for enrollment in the study, by either direct contact of graduates from those universities or by social networks. The inclusion criteria were being a graduate from school of medicine from a Jordanian university within the last three months preceding collection of the questionnaires; in order to assess knowledge soon after graduation before gaining any additional information from experience at work. The questionnaire was distributed personally in paper form and online using google forms. A statement explaining the aim of the study, benefits, and general description was placed on the cover page of the questionnaire; completion of the questionnaire was considered consent for participation.

## Development of the questionnaire

The questionnaire was developed in English since the language used in different forms of teaching in all the medical schools in Jordan is the English language. The questions were established and refined after exhaustive review of literature, including universal textbooks for medical education and international diabetes care guidelines, and medical experience of authors in pediatric endocrinology. After completion of the questionnaire, it was revised by an external researcher, who was an expert in medical education, for the clarity and consistency of the questions. The questionnaire was tested on a pilot sample of 10 participants to ensure clarity of questions, appropriateness of time consumed in filling the questionnaire, and the relevance. Corrections and modifications were preformed accordingly and these responses were excluded from the analysis.

The questionnaire contained two major parts, demographic characteristics and knowledge. Data collected included: gender, name of university, family history of diabetes, number of lectures and seminars about T1D, and approximate number of persons with T1D seen during clinical training. The questions on knowledge were diverse and evaluated different aspects of T1D including pathophysiology, diabetic ketoacidosis, insulin management, hypoglycemia, basic knowledge about using technologies (as glucose continuous monitoring and insulin pumps), basic guidelines for exercise, insulin management in patients planned for surgery, screening for diabetes complications, and types of foods containing carbohydrates.

Questions were with multiple choices; each question had a weight of one point. There was one correct answer and “I do not know” choice, and at least one incorrect answer in each question. The answers were all assessed and analyzed into three groups: correct answer, incorrect answer, and “I do not know”. To calculate the knowledge score, responses that included correct answers were given one point, responses that included incorrect answers or “I do not know” were given zero point. The scores of the 28 questions for each participant were summed into the knowledge score, where 28 was the highest score and zero was the lowest score, and higher scores indicated better knowledge of T1D.

The internal consistency and how closely related the questions on knowledge were assessed with Cronbach’s Alpha, the test score was 0.689.

### Statistical analysis

Statistical analysis was performed using IBM SPSS Statistics for Windows, version 23 (IBM Corp., Armonk, N.Y., USA). Categorical data was presented as frequency (%) and continuous data was described as mean (standard deviation). Comparison of continuous variables among groups was conducted using independent samples T test (between two groups) and one-way ANOVA (between more than two groups). Possible predictors of knowledge score were analyzed using univariate and multivariate linear regression. P values less than 0.05 were considered statistically significant.

## Results

A total of 358 recent medical graduates, out of total 1676 new graduates from the 6 schools of medicine in Jordan, responded to the survey. Female respondents were significantly higher than male respondents, 55.6% and 44.4%, p value = 0.035, respectively. Average number of lectures concerning diabetes during study at the medical school was 3.92 ± 1.37. The general characteristics of the participants are shown in Table 1.

**Table 1:** General characteristics of the participants, N = 358

	Frequency (%)
**Gender**	
Female	199 (55.6%)
Male	159 (44.4%)
**University**	
University A	171 (47.8%)
University B	41 (11.5%)
University C	12 (3.4%)
University D	23 (6.45%)
University E	39 (10.9%)
University F	72 (20.1%)
**Interested in pediatric endocrinology as a future subspecialty?**	
Yes	103 (28.8%)
No	138 (38.5%)
I do not know	117 (32.7%)
**The participant had diabetes?**	
Yes	2 (0.6%)
No	356 (99.4%)
**Family history of diabetes?**	
Yes	236 (65.9%)
No	122 (34.1%)
**Number of persons with type 1 diabetes the participant encountered during the medical years**	
None	9 (2.5%)
Less than 5 patients	86 (24.0%)
Between 5 and 10 patients	134 (37.4%)
More than 10 patients	129 (36.0%)
**Number of lectures on diabetes during medical education years**	
1–2 lectures	73 (20.4%)
3–4 lectures	138 (38.5%)
5–6 lectures	147 (41.1%)
**Expected degree of self-knowledge of diabetes?**	
Poor	61 (17.0%)
Good	272 (76.0%)
Excellent	25 (7.0%)

There was no correlation between the number of lectures attended by the participants during their study years and the knowledge score, Pearson Correlation = 0.078, p value = 0.142. Associations between several variables and the knowledge score were identified, Table 2.

**Table 2:** Correlation between categorical variables of characteristics of the participants and the mean knowledge score

	Mean knowledge score (SD)	P value
**Gender**		
Female	18.78 (3.58)	0.01 ^*^
Male	17.69 (4.22)	
**University**		0.248 ^**^
University A	18.57 (3.72)	
University B	17.81 (4.27)	
University C	19.58 (2.15)	
University D	16.74 (3.98)	
University E	18.03 (2.72%)	
University F	18.36 (4.73%)	
**Interested in pediatric endocrinology as a future subspecialty?**		0.330 ^**^
Yes	17.92 (3.67)	
No	18.24 (3.97)	
I do not know	18.70 (4.05)	
**The participant had diabetes?**		0.128 ^*^
Yes	22.50 (4.95)	
No	18.28 (3.90)	
**Family history of diabetes?**		0.299 ^*^
Yes	18.45 (3.83)	
No	18.00 (4.07)	
**Number of persons with type 1 diabetes the participant encountered during the medical education years**		0.009 ^**^
None	15.33 (3.12)	
Less than 5 patients	17.64 (3.79)	
Between 5 and 10 patients	18.27 (4.02)	
More than 10 patients	18.98 (3.79)	
**Number of lectures on diabetes during medical education years**		0.454 ^**^
1–2 lectures	18.22 (4.27)	
3–4 lectures	18.02 (3.70)	
5–6 lectures	18.60 (3.93)	
**Expected degree of self-knowledge of diabetes**		< 0.001 ^**^
Poor	15.34 (4.18)	
Good	18.85 (3.57)	
Excellent	19.48 (3.62)	

^*^: Independent sample T test, ^**^: One way ANOVA

The percentage of correct answers, incorrect, and “I do not know” were identified for all the questions separately. High percentage of students had correct answers for pathophysiology, certain aspects of diabetic ketoacidosis, hypoglycemia, and screening for diabetes complications, namely autoimmune thyroiditis, Table 3.

**Table 3:** The number and frequency of correct, incorrect, and “I don’t know” answers to the questions of the survey

Questions’ excerpts ^*^	Correct answers N (%)	Incorrect answers N (%)	I do not know N (%)
Q1. Main pathophysiology of type 1 diabetes is …	344 (96.1%)	13 (3.6%)	1 (0.3%)
Q2. Type 1 diabetes can be prevented if investigated early before appearance of symptoms of diabtes …	317 (88.5%)	24 (6.7%)	17 (4.7%)
Q3. More than 90% of persons with type 1 diabetes are inherited …	216 (60.3%)	97 (27.1%)	45 (12.6%)
Q4. Type 1 diabetes can present during the neonatal period …	111 (31.0%)	155 (43.3%)	92 (25.7%)
Q5. Abdominal pain is a known presenting symptom of DKA …	351 (98.0%)	4 (1.1%)	3 (0.8%)
Q6. Which of the following regarding the diagnostic laboratory criteria for DKA is FALSE …	288 (80.4%)	52 (14.5%)	18 (5.0%)
Q7. Regarding first step of management for persons presented with DKA …	318 (88.8%)	40 (11.2%)	0 (0%)
Q8. Regarding insulin management of DKA after first hour of management …	191 (53.4%)	14 (40.5%)	22 (6.1%)
Q9. While a patient was being treated for DKA, he developed sudden decreased level of consciousness, which one of the following may be cause of his condition …	233 (65.1%)	116 (32.4%)	9 (2.5%)
Q10. Usual subcutaneous insulin regimen of persons with type 1 diabetes should include basal and bolus rapid or short-acting insulin …	291 (81.3%)	27 (7.5%)	40 (11.2%)
Q11. If a child with type 1 diabetes has hyperglycemia and it was not meal time…	162 (45.3%)	106 (29.6%)	90 (25.1%)
Q12. Sweating, tremor and palpitations are symptoms of …	302 (84.4%)	50 (14.0%)	6 (1.7%)
Q13. A child with type 1 diabetes had loss of consciousness, his blood glucose was low, the immediate drug to be given is …	304 (84.9%)	29 (8.1%)	25 (7.0%)
Q14. If a child with type 1 diabetes has febrile illness, you expect his blood glucose to …	247 (69.0%)	81 (22.6%)	30 (8.4%)
Q15. A new modality of testing blood glucose other than using glucometer and strips is flash glucose monitoring …	92 (25.7%)	79 (22.1%)	187 (52.2%)
Q16. Insulin pumps are used ONLY for children with type 1 diabetes older than 13 years old …	147 (41.1%)	63 (17.6%)	148 (41.3%)
Q17. If blood glucose is more than 270 mg/dL in child with type 1 diabetes, he is advised to do exercise to lower his blood glucose …	179 (50.0%)	101 (28.2%)	78 (21.8%)
Q18. Glycated hemoglobin is an indicator of glycemic control over the …	344 (96.1%)	5 (1.4%)	9 (2.5%)
Q19. If a child with type 1 diabetes is scheduled for an elective minor surgery, he should NOT receive the long-acting insulin in the preceding night …	155 (43.3%)	119 (33.2%)	84 (23.5%)
Q20. A child with type 1 diabetes wants to eat a meal and his sugar was 100 mg/dl The dose of rapid acting insulin to be given should be calculated according to carbohydrates content in the meal …	240 (67.0%)	48 (13.4%)	70 (19.6%)
Q21. Screening for associated co-morbidities with type 1 diabetes includes screening for hyperlipidemia at puberty …	194 (54.2%)	83 (23.2%)	81 (22.6%)
Q22. If thyroid function test is normal at diagnosis for a child with type 1 diabetes, there is no need for further t testing later on …	295 (82.4%)	33 (9.2%)	30 (8.4%)
Q23. Diabetic retinopathy screening in children with type 1 diabetes should start after puberty regardless of age at diagnosis …	202 (56.4%)	106 (29.6%)	50 (14.0%)
Q24. Oral hypoglycemic agents like sulfonylurea, can be added to insulin therapy after puberty for persons with type 1 diabetes …	177 (49.4%)	85 (23.7%)	96 (26.8%)
Q25. Bran bread contains carbohydrates …	271 (75.7%)	47 (13.1%)	40 (11.2%)
Q26. Eggs contain carbohydrates …	186 (52.0%)	134 (37.4%)	38 (10.6%)
Q27. Fried meat contains carbohydrates …	151 (42.2%)	175 (48.9%)	32 (8.9%)
Q28. Olive oil contains carbohydrates …	243 (67.9%)	75 (20.9%)	40 (11.2%)

^*^: Full questionnaire is listed in the Additional files section

There were 91 (25.4%) graduates who did not know that hypoglycemia can be a cause of decreased level of consciousness during treating DKA. It was found that 56 (15.7%) graduates did not know the symptoms of hypoglycemia and that 15.1% did not know the accurate management of severe hypoglycemia. Regarding hyperglycemia, 12% thought they cannot give rapid-acting insulin to correct it unless it was given at mealtime, and 18% thought that long-acting insulin should be given to correct hyperglycemia. There were 27.6% of graduates that erroneously answered that an individual with T1D should be advised to do exercise whenever blood glucose is more than 270 mg/dL. As for basic information regarding administering insulin prior to a scheduled surgery, 33.2% mistakenly believed that a patient should not receive long-acting insulin the night preceding a minor surgery and 23.5% did not know whether to give it or not. There were 23.7% of graduates who thought that sulfonylurea can be added to insulin for treating adolescents with T1D.

Possible predictors of knowledge were assessed using linear regression. In the multivariate model, two variables were statistically significant, gender and expected degree of self-knowledge of diabetes. Knowledge score of male medical graduates was significantly lower than their female colleagues. Compared to females, male graduates had a lower knowledge score of 1.099, p value = 0.008. Participants were able to evaluate the extent of their knowledge of T1D. Compared to participants who identified themselves with poor knowledge, those who evaluated themselves with good or excellent knowledge had an increase in the knowledge score of 3.223 and 3.924, p values = < 0.001 and < 0.001, respectively, Table 4.

**Table 4:** Possible predictors of the knowledge score using univariate linear regression and multivariate linear regression

	Univariate linear regression			Multivariate linear regression		
	*B*	95% CI	P value	*B*	95% CI	P value
**Gender**						
Female ^*^						
Male	-1.092	-1.903 - -0.281	0.008	-1.099	-1.910-0.288	0.008
**University**						
University A ^*^						
University B	-0.768	-2.103 - o.566	0.258	0.012	-1.421–1.445	0.986
University C	1.010	-1.281–3.302	0.387	0.846	-1.449–3.141	0.469
University D	-1.834	-3.538 - -0.130	0.035	-0.980	-2.667–0.708	0.254
University E	-0.547	-1.909–0.814	0.430	-0.042	-1.340–1.256	0.949
University F	-0.212	-1.290–0.866	0.699	-0.075	-1.121–0.971	0.888
**Interested in pediatric endocrinology as subspecialty**						
Yes ^*^						
No	0.317	-0.684–1.318	0.534	0.842	-0.149–1.832	0.096
I do not know	0.779	-0.260–1.817	0.141	0.972	-0.023–1.966	0.055
**The participant has diabetes?**						
Yes ^*^						
No	-4.225	-9.669–1.219	0.128	-3.598	-8.960–1.764	0.188
**Family history of diabetes?**						
Yes ^*^						
No	-0.553	-1.410 -0.304	0.205	-0.214	-1.032–0.603	0.607
**Number of persons with type 1 diabetes the participant encountered during the medical education years**						
None ^*^						
Less than 5 patients	2.306	-0.356–4.968	0.089	1.219	-1.342–3.779	0.350
Between 5 and 10 patients	2.935	0.319–5.552	0.028	1.757	-0.777–4.290	0.174
More than 10 patients	3.643	1.024–6.263	0.007	1.979	-0.588–4.545	0.130
**Number of lectures on diabetes during medical education years**						
1–2 lectures ^*^						
3–4 lectures	-0.197	-1.311–0.916	0.728	-0.075	-1.266–1.116	0.901
5–6 lectures	0.379	-0.723–1.481	0.499	0.140	-1.132–1.412	0.829
**Expected degree of self-knowledge of diabetes**						
Poor ^*^						
Good	3.509	2.483–4.534	< 0.001	3.223	2.161–4.284	< 0.001
Excellent	4.136	2.417–5.855	< 0.001	3.924	2.113–5.735	< 0.001

^*^: Reference; *B*: Beta coefficient

## Discussion

In general, this study showed deficiencies in certain areas of knowledge of T1D among new medical school graduates. These gaps of knowledge were identified in many studies that evaluated the awareness and profeciencies of different healthcare providers such as physicians and nurses [15, 17, 18].

Female graduates had higher knowledge scores than males, p value = 0.01. These gender differences in medical students were detected regarding opportunities in their career [19], study habits [20], and lack of equity [21]. In theory, compared to their male colleagues, feeling the pressure to excel and be distinguished to obtain opportunities might drive female graduates to improve their knowledge and clinical skills. It might also be due to the possibility that they come from more advantaged background, but socioeconomic status was not collected in our study.

There was no significant difference in the knowledge scores among the different universities, p value = 0.248. Medical schools in Jordan do not have uniform curricula; discripancies in endocrinology education is highly possible. Long et al. revealed inconsistancies in endocrinology education in the curricula in preclinical years in United States medical schools [22]. In the United Kingdom, Smith et al. revealed that training doctors were not confident in dealing with diabetes issues, and differences existed depending on the geographical location, which demands further improvement and standardization of education [23].

Participants with diabetes did not have different knowledge score compared to those without diabetes, p value = 0.128. On the contrary, Kwiendacz et al. study that was conducted on Polish medical students showed that those with T1D obtained the best scores of knowledge [24]. This discripancy between the two studies can be justified by the differences in sample size and the frequency of participants with diabetes.

In Jordan, the number of pediatric endocrinologists who have the knowledge and experience in providing care for children and adolescents with T1D, is scarce. Most children with T1D are seen by pediatricians, internists, general practitioners and to a lesser extent by adult endocrinologists. Since physicians, other than pediatric endocrinologists, are responsible for the management of most children with T1D in many developing countries [25], it is important that they gain adequate knowledge of T1D during their study at medical school. This can reduce the knowledge gap as compared to pediatric endocrinologists and hopefully would result in better diabetes care delivered for children with T1D. The finding of better knowledge scores for graduates, who had encountered higher number of patients with diabetes during medical school, highlighted the importance of practical clinical experience and involvement in the management of persons with T1D to enrich their knowledge.

Hypoglycemia in individuals with diabetes is a critical issue from the perspectives of the patient and the health care provider [26], which requires immediate management to prevent devastating sequelae. It was alarming that 15.7% graduates did not know the classical symptoms of hypoglycemia, and similar percentage did not know how to manage severe hypoglycemia.

Another emergency condition that may occur in individuals with T1D is DKA, which is the main cause of mortality and hospitalization for these patients, its management is complicated and multifaceted [27]. Recent graduates in our study had a fairly good knowledge on the diagnosis and initial management of DKA, but less knowledge for management beyond the initial phase. Most new graduates (98.0%) knew possible presenting symptoms of DKA like abdominal pain, which is reassuring as sometimes diagnosis of this serious condition might be delayed when patients present with non-specific symptoms as abdominal pain. On the other hand, it was worrisome to find that 11.2% of graduates did not know type of fluid bolus that should be given during the first hour of DKA management; they answered either intravenous glucose saline or intravenous sodium bicarbonate. Although this was relatively low percentage, but it should be taken into consideration that it is a critical mistake which may endanger the patient’s life. Much higher percentage (46.6%) did not know insulin management in DKA, they thought they should give intravenous insulin bolus before starting intravenous insulin infusion and others thought they use subcutaneous insulin injections instead of intravenous infusion. Medical graduates should have adequate knowledge of managing this emergency condition guided by the international guidelines [27], especially that emergency rooms in many health care settings in low-resource countries, are attended usually by general practitioners or trainee doctors.

Weakness in the knowledge of certain aspects of DKA management and other aspects of diabetes, in general, was identified in many countries in the region such as Saudi Arabia [28, 29]. Appropriate awareness of DKA diagnosis and management by physicians, would had significant impact on incidence of delayed diagnosis of DKA and better outcomes; mainly in countries with limited resources [30].

Unsurprisingly, basic knowledge was lacking for the use of technologies in diabetes. Only 25.7% knew about new modalities of glucose testing as continuous glucose monitoring. Also 57.9% did not know that insulin pump can be used for children at any age. This may be due to relatively uncommon use of these technologies in Jordan, as unfortunately, those are not currently covered by insurance. The positive effects of continuous glucose monitoring on glycemic control which had been previously reported [31]; necessitates that physicians should have background knowledge about it, so they can encourage its use by patients who can afford it until, hopefully, would be covered by insurance.

There were 247 (69.0%) graduates who knew that blood glucose levels usually increase during febrile illnesses. Physicians caring for children with T1D must be aware of basics of management during intercurrent illnesses [32].

It is alarming that 18.7% did not know the subcutaneous insulin regimens used for T1D patients, and 33% did not know that determining amount of carbohydrates in meals is needed to calculate doses of rapid-acting insulin. Children and adolescents diagnosed with T1D and their parents should be educated shortly after diagnosis on how to calculate insulin doses based on determining the amount of carbohydrates in meals and on blood glucose readings [33]. Basic knowledge of types of food containing carbohydrates ranged between 42.2 and 75.7% for a number of food items. In many developing countries, there is scarce number of experienced dieticians in T1D, and in many health care settings, physicians are responsible primarily to educate patients how to determine insulin doses [34]. Therefore, medical graduates in these countries should have at least basic knowledge about food items that contain carbohydrates.

Awareness about screening for diabetes complications including autoimmune thyroiditis, diabetic retinopathy and hyperlipidemia was variable, 82.4%, 56.4% and 54.2%, respectively. Better knowledge about universal guidelines for screening is extremely important, to ensure earlier diagnosis and hence earlier intervention [35].

The relatively scarce number of pediatric endocrinologists; in the face of rising numbers of children with T1D in developing countries, necessitates modification of curricula of medical school in those countries to mend the gap.

The traditional curriculum that is offered to medical students where there is limited clinical experience confines the development of clinical skills. Additional and versatile types of educational methods should be implemented and studied to fill the gaps in knowledge and clinical practice. In Germany, medical students were not satisfied with the education they received on type 1 and type 2 diabetes, especially clinical experience [36].

Proposed strategies must be carefully introduced and evaluated for its sustainability and effectiveness. Single seminars on diabetes had only short term benefits but lacked durability [37]. Positive improvements were also identified after interactive training session for medical students on hypoglycemia. However, these effects were also assessed shorly after the sessions in form of a pre and post surveys [38]. MacEwen et al. evaluated a “Diabetes Acute Care Day” that included lectures on basic diabetes knowledge, emergencies, management and monitoring. Using a pre and post course scores, the researchers found an increase in all areas of knowledge [39].

Postgraduate courses for general practitioners and senior medical students were also suggested as possible solutions for deficiencies in knowledge [40].

All the educational interventions to improve the knowledge must be assessed for its long-term effect and its impact of on patient care. Meyers et al. evaluated the impact on patient outcomes of a new elective, SPECIAL (Students Providing Education on Chronic Illness and Lifestyle) that was introduced to first year medical students in United States. Patients reported positive experience and most of them had a reduction in their glycated hemoglobin levels, which supports the importance of early introduction to clinical exposure in medical education [41].

Interprofessional education that is gobaly implemented in health care professions is a noteworthy strategy for improving knowledge. An educational tool that was introduced to junior doctors, nurses, healthcare assistants and pharmacists in a hospital setting led to improvement of knowledge and reduction in management errors [42]. In addition, changing the traditional way of teaching, implementing innovative teaching methods, and focusing on more interactive learning may motivate students to actively engage in the education process [43].

Participants perception of their knowledge predicted their knowledge scores. This indicated that medical students and graduates can assess their competencies and accurately identify areas that are deficient and requires improvement. Consequently, elective courses that are diverse and cover several clinical experential education levels should be offered to medical students and tailored depending on their needs.

Based on the results of our study, which showed the importance of increasing clinical exposure of medical students to children with T1D and to be involved in delivering care to them, we are planning to modify our pediatric curriculum for the fifth and sixth year medical students. As a start for the upcoming academic year; we had designed two interactive teaching sessions during students’ rotation in the pediatric department. We used to give seminars during the past years about T1D that included basics about pathophysiology, diabetic ketoacidosis, types of insulin regimens and screening for microvascular and macrovascular complications. We plan for the new interactive teaching sessions, involving case-based teaching focusing on approach for newly diagnosed children with T1D, including management of acute complications, namely hypoglycemia and DKA. In addition to increase exposure during the pediatric clinical rotation to management of children with T1D, and how to counsel patients and their parents shortly after diagnosis, and basics of dietary education and new technologies used in diabetes. We are also planning in the near future to design an interactive case-based workshop for new interns and junior pediatric residents involving basics of T1D diagnosis and management.

Our study included graduates from all the six medical schools in Jordan, but we had the limitation of not having proportionate numbers of respondents from those universities, as 47.8% of respondents were from one university, which is located in the capital city. A higher number of graduates from other medical schools would have been more representative of graduates’ knowledge of T1D in Jordan. Future research with larger sample size representing equally all medical schools in the country; would be useful to set a background for curriculum modification with aim to unify diabetes education in the curricula of the different medical schools in the country. Further studies also would be needed to investigate the effect of the modified curriculum of pediatrics concerning T1D that we are planning to implement, based on the results of our research.

## Conclusion

Conducting studies that evaluate the knowledge of new medicine graduates is essential to evaluate current curricula, experiential eductaion, and clinical experience offered to medical students. Identifying gaps and possible predictors of poor knowledge will help members of educational boards, and policy makers to develop and implement changes in the curricula and promote requirements for graduation. These modifications will enhance knowledge in areas where deficit is detected and reflect on the quality of patient care and health care services provided to individuals with type 1 diabetes.

## Electronic supplementary material

Below is the link to the electronic supplementary material.

**Additional files:** Questionnaire of Medical Graduates knowledge about Type 1 Diabetes

## Data Availability

The datasets analyzed during the current study available from the corresponding author on reasonable request.
