# Supplementary material for: Adequacy of knowledge of new medical graduates about diagnosis and management of children and adolescents with type 1 diabetes in a developing country
Source: BMC Med Educ. 2023 Apr 12;23:234. doi: 10.1186/s12909-023-04234-z (PMC10100482; doi:10.1186/s12909-023-04234-z)
Supplement: Supplementary file 1 — Additional files: Questionnaire of Medical Graduates knowledge about Type 1 Diabetes [file 12909_2023_4234_MOESM1_ESM.pdf]

knowledge of Type 1 Diabetes Questionnaire:
Information about participant:
A. Participant's Name:
B. Gender: ○ Male ○ Female
C. Year of Graduation:
D. University of graduation:
E. Number of lectures about type 1 diabetes/diabetic ketoacidosis during medical study:
F. Approximate number of patients with type 1 diabetes you saw during your clinical rotation at school of medicine: ○ < 5 ○ 5-10 ○ > 10 ○ None
G. Are you interested in endocrinology or pediatric endocrinology as subspecialty in the future: ○ Yes ○ No ○ I don't know yet
H.Do you have Diabetes ? ○Yes ○ No
I. Is there history in your family of any type of diabetes: ○ Yes ○ No
Mark the correct answer:
1.Main pathophysiology of type 1 diabetes is :<br>○ Autoimmune ○Insulin resistance ○ Channelopathies ○ I don't know
2.Type 1 diabetes can be prevented if investigated early before appearance of symptoms of diabetes: ○ Yes ○ No ○ I don't know
3. More than 90% of persons with type 1 diabetes are inherited:<br>○ Yes ○ No ○ I don't know
4. Type 1 diabetes can present during the neonatal period:<br>○Yes ○ No ○ I don't know
DKA (Q5-Q9):
5. Abdominal pain is a known presenting symptom of DKA:<br>○ Yes ○ No ○ I don't know
6. Which of the following regarding the diagnostic laboratory criteria for DKA is <u>FALSE</u>:<br>○PH less than 7.30 ○ Serum bicarbonate 18-20 mEq/L<br>○Positive serum and/or urine ketones ○ I don't know
7. Regarding first step of management for persons presented with DKA in emergency room:<br>○Give intravenous normal saline bolus ○Give intravenous glucose saline bolus<br>○Give Intravenous sodium bicarbonate ○ I don't know
8. Regarding insulin management of DKA after first hour of management:<br>○Start intravenous insulin bolus ○Start continuous insulin infusion without insulin bolus<br>○Start subcutaneous rapid-acting insulin injections ○I don't know
9. While a patient was being treated for DKA, he developed sudden decreased level of consciousness, which one of the following may be cause of his condition:<br>○ Cerebral edema ○ Hypoglycemia ○ A and B ○ I don't know
10. Usual subcutaneous insulin regimen of persons with type 1 diabetes should include basal (long or intermediate-acting insulin) and bolus rapid or short-acting insulin:<br>○ Yes ○ No ○ I don't know
11. If a child with type 1 diabetes has hyperglycemia and it was not meal time:<br>○Give rapid-acting insulin injection ○ Give long-acting insulin injection<br>○ No insulin should be given if it is not meal time ○ I don't know
12. Sweating, tremor and palpitation are symptoms of :<br>○Hyperglycemia ○Hypoglycemia ○ Diabetic ketoacidosis ○ I don't know

**13. A child with type 1 diabetes had loss of consciousness, his blood glucose was low, the immediate drug to be given is:** ○ Glucagon ○ Epinephrine ○ Diazepam ○ I don't know
**14. If a patient with type 1 diabetes has febrile illness, you expect his blood glucose to:** ○ Increase ○ Decrease ○ Not changed ○ I don't know
**15. A new modality of testing blood glucose other than using glucometer and strips is flash glucose monitoring for 24 hour monitoring on demand ex. Freestyle libre:** ○ Yes ○ No ○ I don't know
**16. Insulin Pumps are used ONLY for children with type 1 diabetes older than 13 years old:** ○ Yes ○ No ○ I don't know
**17. If blood glucose is more than 270 mg/dL in a child with type 1 diabetes , he is advised to do exercise to lower his blood glucose:** ○ Yes ○ No ○ I don't know
**18. Glycated hemoglobin (HbA1c) is an indicator of glycemic control over the :** ○ Last one week ○ Last one month ○ Last 3 months ○ I don't know
**19. If a child with type 1 diabetes is scheduled for an elective minor surgery of less than 1 hour long, he should NOT receive the long acting insulin (glargine) in the preceding night as he will be fasting for the surgery (NPO):** ○ True ○ False ○ I don't know
**20. A child with type 1 diabetes wants to eat a meal and his blood glucose was 100 mg/dl (5.5 mmol/L). The dose of rapid acting insulin to be given should be calculated according to carbohydrates content in the meal:** ○ Yes ○ No ○ I don't know
**21. Screening for associated co-morbidities with type 1 diabetes includes screening for hyperlipidemia at puberty:** ○ Yes ○ No ○ I don't know
**22. If thyroid function test is normal at diagnosis for a child with type 1 diabetes, there is no need for further testing thyroid function later on:** ○ True ○ False ○ I don't know
**23. Diabetic retinopathy screening in children with type 1 diabetes should start after puberty regardless of age at diagnosis of type 1 diabetes:** ○ Yes ○ No ○ I don't know
**24. Oral hypoglycemic agents like sulfonylurea, can be added to insulin therapy after puberty for patients with type 1 diabetes:** ○ Yes ○ No ○ I don't know
**Carbohydrates:**
**25. Bran bread contains carbohydrates:** ○ Yes ○ No ○ I don't know
**26. Eggs contain carbohydrates:** ○ Yes ○ No ○ I don't know
**27.Fried meat contains carbohydrates:** ○ Yes ○ No ○ I don't know
**28. Olive oil contains carbohydrates:** ○ Yes ○ No ○ I don't know
**In general, how do you describe your knowledge about diagnosis and management of type 1 diabetes:** ○ Excellent ○ Good ○ Poor
**END of Questionnaire**<br>**Thank you so much for your cooperation**
